# Recognizing Innovation in Academic Advancement

**DOI:** 10.1016/j.jacadv.2025.101704

**Published:** 2025-04-25

**Authors:** Kartik Agusala, Krishna Pundi, Anjali Thakkar, David Cho, Eric D. Peterson, Ami Bhatt, Jennifer N. Avari Silva

**Affiliations:** a University of Texas Southwestern Medical Center, Dallas, Texas, USA; b University of California-San Francisco, San Francisco, California, USA; c Palo Alto Foundation Medical Group, Palo Alto, California, USA; d University of California-Los Angeles, Los Angeles, California, USA; e Harvard Medical School, Boston, Massachusetts, USA; f Washington University School of Medicine, Washington University in St. Louis, St. Louis, Missouri, USA

**Keywords:** academic advancement, innovation, promotion

Faculty promotion at academic medical centers (AMCs) has focused primarily on clinical care, research, and education, as measured by traditional work productivity and teaching metrics, peer-reviewed publications, and grants. However, an increasing number of academic faculty are engaging in innovation-related activities in the areas of information technology, medical devices, and health care delivery. These physicians leverage their knowledge and creativity to address new, complex health care challenges and bridge the gap between clinical practice, technology, and industry in order to benefit medicine and society at large. The ever-expanding role of health care innovation in academia demands the recognition of efforts beyond traditional metrics. Moreover, a recent study by Ligibel et al noted that 32% of physicians within an AMC were considering leaving within 2 years and that measures of higher burnout and lower professional fulfillment were associated with greater intention to leave.^1^ We believe that these faculty and their efforts need to be acknowledged, valued, and incentivized, not only for their career development and well-being but also to maximize the success of AMCs and their innovation efforts across the country. To successfully promote this mission and create a more rewarding and fulfilling professional environment, we propose that AMCs incorporate nontraditional metrics that formally recognize and reward physicians for innovation-related work in the academic advancement and promotion process. Specifically, a core set of universal innovation metrics should be developed across leading AMCs and professional societies to serve as a framework for each institution to modify and adapt to its own needs.

Clinical health care innovation is an increasing focus of AMCs and their faculty, particularly as they explore novel paths of development and commercialization beyond traditional patents and licenses. At its core, clinical innovation of high-quality products and services represents an extension of the tripartite academic mission of clinical care, research, and education and confers multiple benefits to AMCs. Successful commercialization of technologies developed within AMCs generates revenue, leads to the formation of new companies, and creates wider institutional recognition and brand awareness. These advances improve health care value, operational efficiency, and outcomes while expanding patient access and reducing costs. Importantly, clinical faculty are uniquely positioned to innovate given that patient care involves numerous challenges and inefficiencies that create opportunities to create high-value products and services to address unmet needs. By formally recognizing and rewarding faculty for their work in this space, AMCs will advance the mission of clinical innovation, maximize the success of academic innovation hubs, increase faculty retention, and reduce burnout. Innovation metrics can be broadly categorized by their focus, either externally with entrepreneurship metrics or internally with intrapreneurship metrics. Given the significant variability in clinical innovation programs nationally, these innovation metrics should be tailored by each AMC to best support the scope and goal of its individual mission. There are also multiple other future and long-term possibilities to consider in this shift toward valuing and promoting clinical innovation in the academic advancement process.

## Entrepreneurship metrics

In today's evolving landscape, academic faculty are frequently involved in the entrepreneurship and health technology landscape in several roles, ranging from consultants or scientific advisors to research partners or founders. These roles often require significant time commitment and result in scholarly output yet are not formally recognized in the academic advancement and promotion process. A key barrier to academic recognition for entrepreneurial activities is the lack of appropriate guardrails to mitigate conflict of interest issues that may arise from competing financial incentives and bias in research activities. We propose metrics for both assessing success in entrepreneurial work and establishing guardrails to minimize conflict of interest.

### Metrics for assessing entrepreneurial success

• Generation of intellectual property (IP): Commercialization of academic innovation often involves obtaining patents and then licensing the protected innovation in exchange for cash or equity. Tracking the number of patents disclosed, submitted, and granted provides a quantitative metric of entrepreneurial productivity and success. Copyrights, another form of IP protection, are often used to protect software and can also be readily measured.
• License agreements and revenue generation: License agreements, the primary method of commercialization in academia, and their associated revenue can serve as a clear metric of the impact and success of an invention and the benefit conferred to the institution. Revenue generation outside of licenses, such as subscription sales, can also be tracked.
• Industry-sponsored research agreements and associated scholarly output: Industry-sponsored research, including clinical trials funded by an external company or organization, is an important mechanism that advances the academic research mission. Tracking the number of industry-sponsored clinical trials a faculty member has participated in, their specific role, and the associated scholarly output (publications, conference presentations) can serve as a valuable metric of academic output that is analogous to traditional, grant-funded clinical trials.
• Funding: Whereas traditional academic funding is primarily government or nonprofit funded, funding associated with entrepreneurship can take multiple forms. Aside from NIH Small Business Innovation Research and Small Business Technology Transfer grants, most entrepreneurial funding is privately sponsored. Tracking amounts of venture or dilutive funding and industry-sponsored grants and awards can provide an entrepreneurial correlate to traditional academic grant funding.
• Roles: Health system leadership roles are rewarded in the academic advancement process, yet leadership roles in companies or external organizations are not similarly recognized. One stated rationale for this discrepancy is that external roles are generally compensated with cash or equity. However, the same is true for many internal health system leadership roles, which often protect a percentage of the faculty's salary. Therefore, AMCs should consider tracking and recognizing the number and type of leadership roles in entrepreneurial organizations, ranging from advisory roles to product management roles to key leadership roles.
• Mentorship: Just as in academia, mentorship is a core component to entrepreneurial success. Successful mentorship and guidance provided to entrepreneurs, inventors, and start-up companies should be rewarded and can have downstream positive effects such as incentivizing those with robust industry connections to open their networks and help democratize access to industry.
• Product development: Certain types of products and services, such as digital health applications, are often not reflected in the above metrics but require significant time and effort and provide value. AMCs may choose to recognize these innovations, either upon completion or in a graded fashion based on stages of development.
• Venture companies: Establishing an independent company is an important milestone along the commercialization pathway that accrues multiple benefits to AMCs and should be valued as a distinct innovation metric.

### Mitigating conflict of interest between entrepreneurial pursuits and academia

• IP Management: Development and enforcement of agreements that clearly define IP ownership and commercialization rights are important and should be facilitated by the AMC's commercialization and technology transfer office to ensure fairness and standardization.
• Resource Allocation: AMCs need to clearly delineate expectations around faculty time and effort commitments to avoid the compromise of academic and clinical responsibilities. Institutions should define acceptable and reasonable faculty time limits for industry-related activities. Any use of institutional resources must be clearly stated and agreed upon upfront, including cost-sharing guidelines, if applicable.
• Academic Freedom: Institutions should establish policies that protect the right to publish research findings developed by faculty without industry restriction. Faculty-industry contracts should be reviewed by institutions to ensure the presence of clauses that safeguard academic autonomy.
• Public Reporting/Disclosure: Developing a frequently updated and publicly searchable registry disclosing all faculty industry relationships and financial interests can promote transparency.

## Intrapreneurship

Intrapreneurship refers to innovation, product development, and process improvement within an organization. Intrapreneurs leverage their deep understanding of institutional structure to advance their organizations, and their impact can be measured by effects seen on health care quality and outcomes, cost savings, patient access, reduction of inequities, patient satisfaction and overall growth. Such contributions should be recognized in the academic advancement process, and metrics of intrapreneurship success can be assessed in the following 5 categories:

• Product and Process Development: Clinician innovators leverage their unique patient care perspective and working institutional knowledge to create novel products and processes that enhance all aspects of the care paradigm. Such improvements include information technology and application development, enhancements to the electronic medical record, internal think tanks and steering groups to improve workflow, and a wide variety of quality improvement initiatives. AMCs can decide whether such interventions should be recognized while still in process or only upon completion, with additional consideration for those interventions that have resulted in tangible improvements.
• Delivery of Care: Intrapreneurs can develop novel pathways and process frameworks to optimize the use and deployment of clinical resources. Examples may include virtual group patient visits or counseling, establishment of multidisciplinary clinics structured around a specific disease, or optimization of specialty referrals to reduce delays in care. Faculty that pioneer and implement such changes should be given formal recognition for their involvement in such initiatives.
• Leadership and Mentoring: Leaders and mentors who foster an organizational growth mentality and empower innovation through their work and in their mentees are crucial for the sustained success of any organization. An intrapreneur's impact here can be measured through the number of their mentees and the productivity and success of those mentees. Institutions should also reward leaders who demonstrate managerial innovation by tracking new programs and initiatives that enhance employee motivation, well-being, problem-solving, and communication.
• Health Care Economics: Innovation in revenue generation and provider payments, including value-based care models or quality-based incentives, can lead to higher value of care by limiting the utilization of high-cost, low-value tests and interventions. Individuals who champion such changes should be recognized for their contribution to the institution and the health care landscape.
• Individual Capacity Development: Formal training programs are often costly and incur a significant time commitment. However, these programs are a powerful way for clinicians to expand their capacity and develop new perspectives, all of which ultimately benefit their institutions in the long term. Training and certificate programs, such as in artificial intelligence, clinical informatics, and biodesign, and formal education such as Masters degrees, represent an individual's commitment to scholastic and personal growth that should be formally recognized in the academic advancement process.

## Future and long-term possibilities

For the long term, AMCs should strongly consider creating a new, or parallel, clinical innovator promotion track that would allow faculty innovators a well-defined, accepted career pathway within academia. The proposal of such a pathway was described previously by Majmudar et al,^2^ but ambiguity surrounding standardized metrics and concern that an innovation track would not be viewed with equal weight as traditional tracks limited widespread adoption. Training programs have also recognized the demand and need to develop future clinical innovators, with numerous residency and fellowship programs offering innovation tracks and programs to equip trainees with the fundamental knowledge and skills necessary for future intrapreneurship and entrepreneurship. There has also been a marked increase in dedicated yearlong health technology and innovation fellowships, both within academia and industry that offer valuable education, experience, and networking. Establishing a formal clinical innovator promotion track will provide new graduates and junior faculty with clear direction, resources, and opportunities while validating the continued investment in these popular programs. AMCs can define the metrics, deliverables, and goals of such a track in a manner that best supports their institutional and academic mission.

Quantifiable entrepreneurial and intrapreneurial metrics that recognize and reward the accomplishments of physician innovators is a much-needed next step to encourage this important career path within academic medicine. AMCs can tailor these metrics to align with their strategic mission and vision by incorporating the broad framework described above. We anticipate that those AMCs that embrace physician innovators and craft academic measures of success will be rewarded in both the short and long term.

## Funding support and author disclosures

Dr Pundi has received consulting fees from iRhythm, Inc and Evidently, Inc that are not relevant to this topic. Dr Avari Silva is the co-inventor and co-founder of SentiAR, Inc and Excera, Inc (both companies have licensed intellectual property from Washington University); and acts as a consultant to both companies. Dr Peterson has received research support from 10.13039/100002429Amgen and 10.13039/501100022336Esperion; has received support from Novo Nordisk and Janssen Pharmaceuticals for serving on their executive trial committees; and support for the DSMB chair for an Edwards Lifesciences trial. All other authors have reported that they have no relationships relevant to the contents of this paper to disclose.
